# High throughput screening data for a case study of CHO cell culture process development

**DOI:** 10.1016/j.dib.2021.107491

**Published:** 2021-10-17

**Authors:** Qin He, Jianlin Xu, Erik Vandermark, Jun Tian, Yueming Qian, Cynthia Chelius, Jongchan Lee, Michael C. Borys, Zheng Jian Li, Girish Pendse

**Affiliations:** a Biologics Development, Global Product Development and Supply, Bristol Myers Squibb, Devens, MA 01434, USA; b Biologics Development, Global Product Development and Supply, Bristol-Myers Squibb, Summit, NJ 07901, USA

**Keywords:** CHO cell culture, High-throughput screening, Rosmarinic acid, Monoclonal antibody titer

## Abstract

In this article, we present four sets of data from high-throughput screening (HTS) studies of different chemically defined media using an industrially relevant Chinese hamster ovary (CHO) cell line. While complex hydrolysate media was used in the early phase process development and manufacturing of a monoclonal antibody (mAb), here we seek to determine an appropriate chemically defined media for late phase process development. Over 150 combinations of chemically defined basal media, feed media, and basal and feed media supplements, such as polyphenolic flavonoid antioxidants (including rosmarinic acid (RA)), were evaluated in four HTS studies to replace the complex hydrolysate media. Specifically, these four screening studies incorporated custom design of experiment (DOE), one-factor-at-a-time (OFAT), and definitive screening design methodologies for titer improvement. Titer was improved two fold compared to the early phase process using the addition of RA to chemically defined media. This dataset exemplifies how HTS can be used as an effective approach to systematically and statistically determine media composition and supplementation to increase mAb titer. These data were presented in connection with a published paper [1].

**Specification Table**

**Table:** 

Subject	Biotechnology
Specific subject area	Improving mAb titer for CHO cell culture by media optimization using HTS
Type of data	Tables Figures
How data were acquired	JMP Statistic software (SAS) Used in the design and data analysis of HTS fed-batch production cultures. HTS fed-batch cell culture HTS fed-batch cell culture operation was performed in 50-mL TubeSpin bioreactors within an incubator (Infors HT, Bottmingen, Switzerland) and Tecan liquid handler (Tecan, Männedorf, Switzerland) for inoculation, sampling, and feeding operations. Titer Titer data were acquired from day 14 HTS culture supernatant samples and subsequent elution through a Protein A affinity column (POROS A20 2.1 × 30 mm column, Life Technologies Corporation, Bedford, MA) by HPLC (Waters, Milford, MA) [2]. The eluted mAb titer was measured by absorbance at 280 nm and quantified by comparing with the reference standard.
Data format	Analyzed
Parameters for data collect	Cell culture samples from each condition of four HTS experiments (in duplicate, 441 total) were collected on day 14 for titer testing
Description of data collection	For day 14 samples, 10 mL of sample was centrifuged at 1000 × g for 10 min, filtered, and purified by HPLC using Protein A affinity columns for titer assay.
Data source location	Bristol Myers Squibb, Syracuse, New York, United States of America
Data accessibility	With the article
Related research article	Jianlin Xu, Matthew S. Rehmann, Jun Tian, Qin He, Jie Chen, Jongchan Lee, Michael C. Borys, Zheng Jian Li, (2020) Rosmarinic acid, a new raw material, doubled monoclonal antibody titer in cell culture manufacturing. Biochem Eng Jrnl, 160: 107637. https://doi.org/10.1016/j.bej.2020.107637

**Value of the Data**

• Provides a roadmap for researchers to conduct a wide range of efficient CHO cell culture media studies using an automated HTS.
• Custom DOE, OFAT and definitive screening designs described in this work will serve as a statistical framework during initial media screening.
• These data detail the impact of various media combinations and media supplements, such as polyphenolic flavonoids, on culture titer and serves as a starting point for optimized CHO cell culture process development.

## 1 Data Description

The data included in this article provide a more in depth look at efficient and effective HTS media development strategies for a late phase process development using CHO cells. This article details different media types and key media components that had positive effects, such as polyphenolic flavonoid antioxidants [3] on cell culture titer.

CHO cell culture titer is a critical cellular attribute to leverage in order to improve efficiency of mAb production processes and reduce manufacturing costs. Titer data were collected for two CHO cell lines expressing the same mAb during fed-batch production culture with over 150 combinations of media types and additives. To improve process development efficiency, we used an automated HTS and employed custom DOE, OFAT, and definitive screening designs.

The first HTS run (e.g., HTS1) was designed using a custom DOE (SAS JMP) with 47 conditions in duplicate. We tested different basal media (B1, B3) and cell line (C1, C2) as different combinations (B1/C1, B1/C2, and B3/C1), three different feed media (F1, F2, F3), and medium components and additives including dextran sulfate, catechin (a polyphenoid flavonoid), iron sulfate, sodium phosphate, and one dry powder medium (DPM) each at three levels (Table 1). The media components and additives were chosen based on previous internal development studies.

**Table 1:** Medium factors and levels tested in HTS1 cell culture using a custom DOE for 47 conditions in 50 mL TubeSpin bioreactors (*n* = 2).

Factors	Level 1	Level 2	Level 3
Basal/Cell Line	B1/Cell line1 (C1)	B1/C2	B3/C1
Feed	F2	F3	F1
Dry powder medium1 (DPM1) in feed	-1	0	+1
Dextran sulfate in feed	-1	0	+1
Catechin in Basal/Feed	-1	0	+1
FeSO4•in Basal	-1	0	+1
Na2HPO4 in feed	-1	0	+1

Levels 1 (-1), 2 (0), and 3 (+1) refer to media components at the baseline level (i.e., no addition), 50% of the maximum, and the maximum concentrations, respectively.

The DOE design conditions and average titer results are shown in Table 2.

**Table 2:** DOE design conditions and average titer results (*n* = 2) for 47 conditions in HTS1.

								Normalized titer		
Run#	Basal/Cell Line	Feed	Catechi*n*	Dextran sulfate	DPM1	Na2HPO4	FeSO_4_	A	B	Mean
1	B1/C2	F2	1	1	1	1	-1	0.84	N/A	0.84
2	B1/C2	F1	1	-1	-1	-1	-1	1.06	1.15	1.10
3	B1/C2	F3	0	0	-1	0	1	0.65	N/A	0.65
4	B1/C2	F2	-1	1	0	1	0	0.90	0.99	0.94
5	B1/C2	F1	-1	-1	0	-1	1	0.98	1.05	1.01
6	B1/C2	F3	-1	0	0	0	-1	0.71	N/A	0.71
7	B1/C2	F1	0	0	0	0	-1	0.98	1.06	1.02
8	B1/C2	F1	1	-1	1	-1	1	0.83	N/A	0.83
9	B1/C2	F2	-1	-1	1	-1	-1	1.03	1.12	1.07
10	B1/C2	F3	1	0	1	1	1	0.64	N/A	0.64
11	B1/C2	F2	1	-1	0	0	0	0.92	N/A	0.92
12	B1/C2	F2	1	1	0	-1	-1	0.97	1.03	1.00
13	B1/C2	F1	1	1	-1	1	1	0.96	0.99	0.97
14	B1/C2	F2	1	1	0	-1	1	0.91	N/A	0.91
15	B1/C2	F2	-1	-1	-1	-1	-1	1.14	1.26	1.20
16	B3/C1	F1	0	0	0	0	1	2.22	2.36	2.29
17	B1/C1	F1	0	0	1	-1	1	3.03	3.13	3.08
18	B1/C1	F3	0	0	1	0	-1	2.69	2.79	2.74
19	B1/C1	F2	0	0	0	1	-1	2.88	2.90	2.89
20	B1/C1	F3	0	-1	-1	1	1	2.74	2.76	2.75
21	B1/C1	F1	1	1	-1	1	0	2.69	2.69	2.69
22	B1/C1	F2	-1	-1	1	0	0	2.16	2.18	2.17
23	B1/C1	F2	0	1	0	1	1	3.10	3.14	3.12
24	B1/C1	F1	-1	1	-1	-1	0	2.37	2.37	2.37
25	B1/C1	F3	-1	0	0	0	0	2.02	2.02	2.02
26	B1/C1	F2	1	-1	1	0	0	3.12	3.14	3.13
27	B1/C1	F1	-1	1	0	1	1	1.45	1.81	1.63
28	B1/C1	F2	1	0	-1	-1	1	2.73	2.77	2.75
29	B1/C1	F3	0	-1	-1	-1	-1	2.68	2.70	2.69
30	B1/C1	F1	-1	0	-1	-1	-1	1.94	2.00	1.97
31	B1/C1	F1	0	1	1	1	1	3.18	3.22	3.20
32	B3/C1	F3	0	-1	0	-1	-1	2.18	2.26	2.22
33	B3/C1	F2	-1	0	0	0	-1	1.03	1.25	1.14
34	B3/C1	F1	1	0	1	1	-1	1.75	2.03	1.89
35	B3/C1	F2	1	1	0	-1	-1	2.67	2.77	2.72
36	B3/C1	F2	0	0	1	1	0	2.72	2.84	2.78
37	B3/C1	F1	-1	0	1	-1	0	0.93	N/A	0.93
38	B3/C1	F1	0	1	-1	1	-1	2.30	2.38	2.34
39	B3/C1	F3	1	0	-1	0	-1	1.90	2.04	1.97
40	B3/C1	F3	-1	1	1	1	1	1.64	1.72	1.68
41	B3/C1	F2	0	0	-1	0	0	2.82	2.82	2.82
42	B3/C1	F2	-1	0	-1	-1	1	1.42	1.46	1.44
43	B3/C1	F3	-1	-1	-1	1	-1	1.05	1.16	1.10
44	B3/C1	F2	1	-1	1	0	1	2.85	3.01	2.93
45	B3/C1	F3	1	1	-1	1	1	2.82	2.84	2.83
46	B3/C1	F3	1	1	1	1	1	2.81	2.89	2.85
47	B3/C1	F2	-1	-1	-1	-1	-1	1.14	1.34	1.24
Control	B0/C1	F0	-1	-1	-1	-1	-1	1.48	1.54	1.51

Level 1 (-1), 2 (0), and 3 (+1) refer to media components at the baseline level (i.e., no addition), 50% of the maximum, and the maximum concentrations, respectively.

**Fig. 1:**
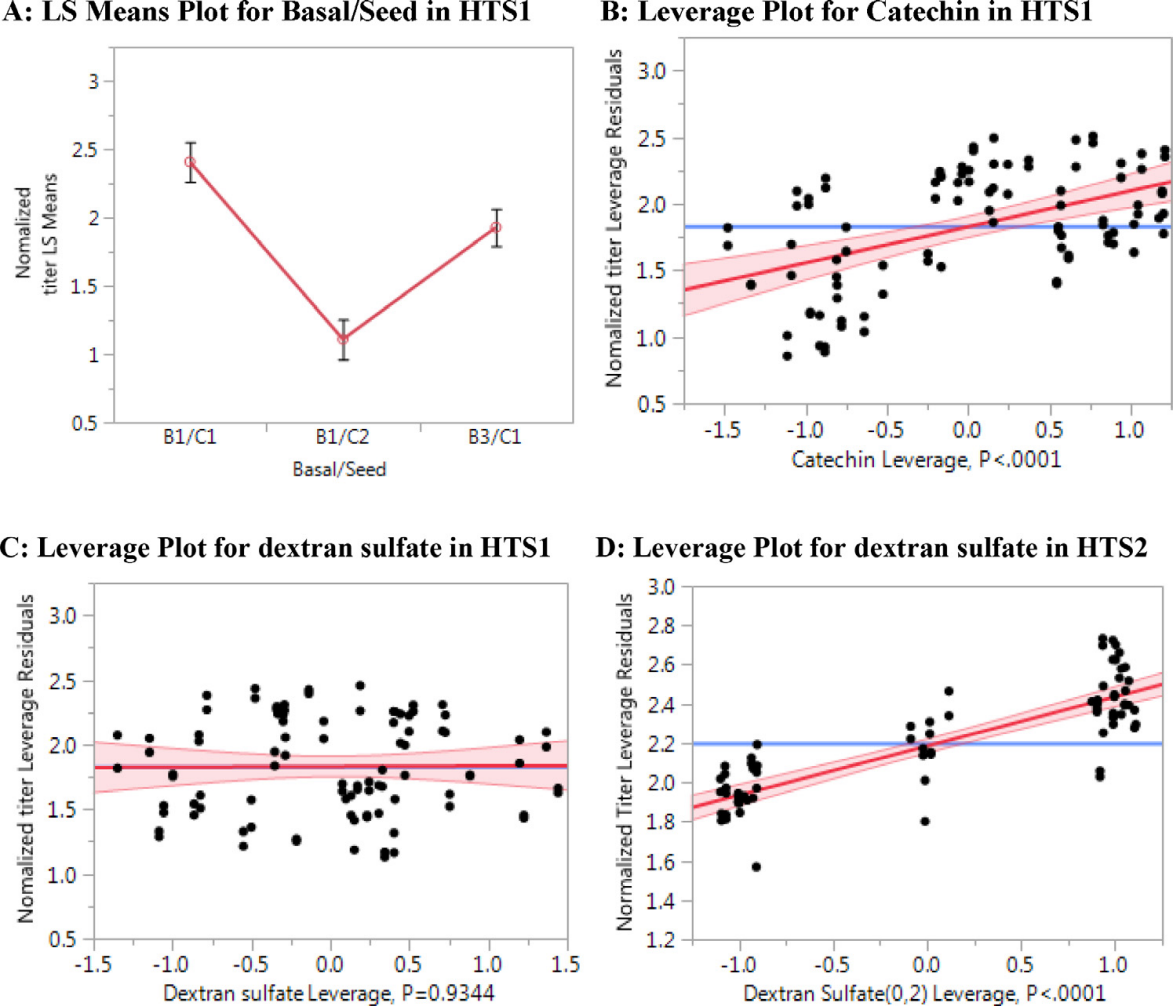
Effects of different factors on mAb titer in HTS1 cell culture (*n* = 2, experimental design in Table 1) and HTS2 cell culture (*n* = 2, experimental design in Table 3): (A) Least Squares (LS) Means Plot for the factor “Basal/Seeds” in HTS1; (B) Leverage plot for catechin in HTS1; (C) Leverage plot for dextran sulfate in HTS1; (D), Leverage plot for dextran sulfate in HTS2.

As shown in Fig. 1A, the condition with the combination of B1 basal and cell line 1 (B1/C1) achieved the highest titer compared to the B1/C2 and B3/C1 conditions. The C1 cell line, which was used in early phase process development and clinical manufacturing, was subsequently selected for the rest of this study. Based on high production titer (Fig. 1A), chemically defined media B1 was selected for further basal medium development.

The top 10 conditions in HTS1 achieved titers between 2.8 and 3.2, which were approximately 2 times higher than the control titer using the early phase process (Table 2). Each of the top 10 conditions contained the polyphenolic flavonoid catechin (Table 2), indicating that catechin was the most important component in HTS1 for increasing titer. This was confirmed in the leverage plot analysis of catechin on titer (*P* < 0.001) (Fig. 1B). In contrast, dextran sulfate did not show a statistically significant effect on titer during HTS1 (Fig. 1C).

Since catechin is a new raw material in cell culture, it was preferred to test existing compounds in the manufacturing raw material list that could be used to increase product titer. Additional additives were screened in HTS2 without any polyphenolic flavonoids (Table 3). All combinations of components in HTS2 were chosen based on previous internal development studies. HTS2 design and average titer results are shown in Table 4. The top 10 conditions had a titer between 2.6 and 2.7 (>70% greater than the early process, but less than the highest producing conditions with catechin in HTS1). In HTS2, each of the top 10 conditions contained dextran sulfate (data not shown), indicating that dextran sulfate was the most important component for titer increase when a polyphenolic flavonoid was not included in HTS2. This was confirmed in the leverage plot analysis of dextran sulfate on titer (*P* < 0.001) in HTS2 (Fig. 1D). The reason dextran sulfate did not positively affect titer in HTS1 (Fig. 1C) was likely due to catechin (Fig. 1B), which would compensate for the effect of dextran sulfate.

**Table 3:** Medium factors and levels tested in HTS2 cell culture using custom DOE for 46 conditions in 50 mL TubeSpin bioreactors (*n* = 2).

Factors	Level 1	Level 2
Spermine in feed F2	-1	+1
Putrescine in feed F2	-1	+1
Ethanolamine in feed F2	-1	+1
Dextran sulfate in feed F2	-1	+1
Tyrosine in feed F2	-1	+1
Asparagine/Lysine/Phenylalanine in feed F2	-1	+1
FeSO4 in Basal B1	-1	+1
Vitamins in feed F2	-1	+1
DPM1 in feed F2	-1	+1

**Table 4.** DOE design conditions and average titer results (*n* = 2) for 46 conditions in HTS2.


										Normalized titer		

Run#	Spermine in feed	Putrescine in feed	Ethanolamine in feed	Dextran sulfate in feed	Tyrosine in feed	Asparagine/ Lysine/ Phenylalanine in feed	FeSO4 in Basal	Vitamins in feed	DPM1 in feed	A	B	Mean
1	-1	1	-1	1	1	-1	1	1	-1	2.52	2.73	2.63
2	1	1	1	-1	1	-1	1	-1	1	2.04	2.20	2.12
3	-1	1	1	-1	-1	-1	-1	-1	1	2.07	2.15	2.11
4	1	-1	-1	1	1	-1	1	1	1	2.42	2.50	2.46
5	-1	1	1	1	-1	-1	1	-1	-1	2.32	2.42	2.37
6	-1	-1	1	1	1	-1	-1	1	1	2.50	2.51	2.50
7	1	1	-1	-1	1	-1	-1	1	1	2.17	2.18	2.18
8	-1	-1	-1	-1	1	1	1	1	-1	2.32	2.45	2.38
9	-1	-1	-1	-1	-1	-1	1	1	1	2.24	2.35	2.29
10	1	-1	-1	-1	1	1	1	-1	1	2.03	2.32	2.17
11	-1	-1	1	-1	1	1	-1	-1	1	2.15	2.37	2.26
12	-1	1	1	-1	-1	1	-1	1	1	2.17	2.30	2.23
13	-1	1	1	-1	1	-1	-1	1	-1	2.21	2.38	2.29
14	1	1	-1	1	1	-1	-1	-1	1	2.37	2.42	2.39
15	1	1	-1	1	1	1	-1	1	-1	2.57	2.62	2.60
16	1	1	1	1	-1	-1	1	1	1	2.42	2.50	2.46
17	-1	-1	1	1	1	1	-1	-1	-1	2.53	2.83	2.68
18	-1	1	1	1	-1	1	-1	1	-1	2.22	2.67	2.44
19	-1	1	1	-1	1	1	1	-1	-1	2.21	2.34	2.27
20	-1	1	-1	1	-1	1	1	-1	-1	2.14	2.57	2.35
21	-1	-1	1	1	-1	1	1	-1	1	2.38	2.50	2.44
22	-1	1	1	-1	-1	-1	1	1	-1	2.09	2.33	2.21
23	-1	-1	1	-1	-1	-1	-1	1	-1	2.08	2.26	2.17
24	-1	-1	1	-1	1	-1	1	-1	-1	2.17	2.48	2.32
25	1	1	-1	-1	-1	1	1	1	-1	2.07	2.44	2.25
26	-1	-1	-1	1	-1	1	-1	1	1	2.47	2.71	2.59
27	1	1	1	1	1	1	1	1	-1	2.61	2.79	2.70
28	-1	1	-1	1	1	1	-1	-1	1	2.46	2.68	2.57
29	1	-1	-1	-1	1	-1	-1	-1	-1	2.07	2.24	2.16
30	-1	1	-1	-1	-1	-1	-1	1	-1	2.14	2.33	2.24
31	-1	-1	1	1	-1	-1	1	1	-1	2.38	2.64	2.51
32	1	1	1	1	-1	1	-1	-1	-1	2.11	2.58	2.34
33	1	1	1	-1	-1	1	1	-1	1	1.95	2.35	2.15
34	-1	1	-1	-1	1	-1	1	-1	1	2.23	2.44	2.34
35	1	-1	1	-1	1	1	-1	1	-1	2.24	2.55	2.39
36	1	1	1	1	1	-1	-1	1	-1	2.50	2.66	2.58
37	-1	1	-1	1	1	1	1	1	1	2.61	2.75	2.68
38	1	-1	1	-1	-1	1	1	-1	-1	2.03	2.19	2.11
39	1	1	-1	-1	-1	-1	1	-1	-1	2.01	2.25	2.13
40	1	-1	-1	-1	-1	1	-1	-1	1	2.10	2.29	2.19
41	1	1	1	1	1	1	-1	1	1	2.62	2.83	2.73
42	1	-1	1	-1	-1	1	1	1	1	2.23	2.46	2.35
43	-1	-1	-1	1	-1	-1	-1	-1	-1	2.43	2.67	2.55
44	1	-1	1	1	-1	-1	-1	-1	1	2.37	2.54	2.45
45	-1	-1	-1	1	1	-1	1	-1	1	2.57	2.58	2.57
46	1	-1	-1	1	-1	-1	-1	1	-1	2.39	2.62	2.50

**Table 5:** Dextran sulfate (DS) sources and polyphenolic flavonoids tested in HTS3 using OFAT design.

Name	Vendor	Cat#
Dextran sulfate: DS1	Sanofi	1176131
Dextran sulfate: DS2	American International Chemicals	DEXSUF
Dextran sulfate: DS3	Sigma	31404
Dextran sulfate: DS4	Sigma	D4911
Dextran sulfate: DS5	Sigma	68076
Dextran sulfate: DS6	Sigma	40357
Dextran sulfate: DS7	Sigma	D6924

Rosmarinic acid	Sigma	536954
	Cayman Chemical	70900
	Spectrum	R1062

Catechin	Sigma	1096790
Resveratrol	Sigma	R5010

**Fig. 2:**
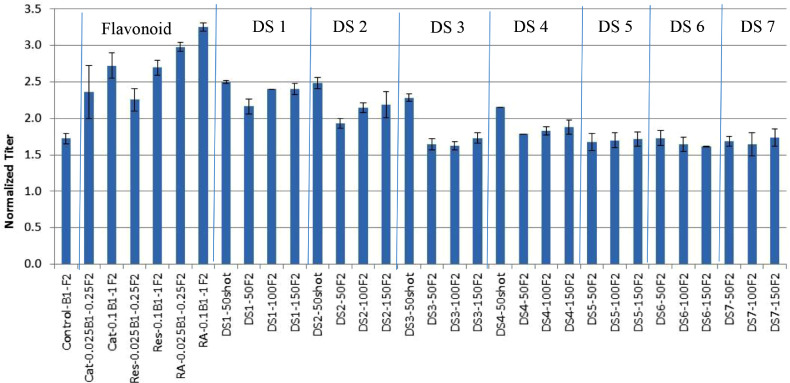
Effects of polyphenolic flavonoids, e.g., catechin (Cat), resveratrol (Res) and RA, and different dextran sulfate (DS) products on the mAb titer in HTS3 using OFAT design with B1 basal and F2 feed (*n* = 2). Control-B1-F2: B1 basal and F2 feed only; Cat-0.025B1-0.25F2: 0.025 mM Cat in B1 and 0.25 mM Cat in F2; Cat-0.1B1-1F2: 0.1 mM Cat in B1 and 1 mM Cat in F2; similar nomenclatures were used for Res and RA conditions; DS1-50shot: 50 mg/L of DS1 was shot into cell culture on Day3; DS1-50F2: 50 mg/L of DS1 was added into F2; DS1-100F2: 100 mg/L of DS1 was added into F2; DS1-150F2: 150 mg/L of DS1 was added into F2. All raw data for HTS3 are presented in Supplemental Table 1.

HTS3 was run as a series of OFAT experiments to compare the effects of seven different dextran sulfate products and three different polyphenolic flavonoids (e.g., catechin, resveratrol, and rosmarinic acid) (Tables 4 and 5). Conditions containing rosmarinic acid achieved the highest titer, outperforming conditions with catechin, resveratrol, or dextran sulfate from different vendors (Fig. 2). Higher concentrations led to slightly higher titer for each of the polyphenolic flavonoids, while a dextran sulfate bolus at 50 mg/L on day 3 led to a higher titer than the conditions with dextran sulfate at 50–150 mg/L added in the feed medium (Fig. 2).

**Fig. 3:**
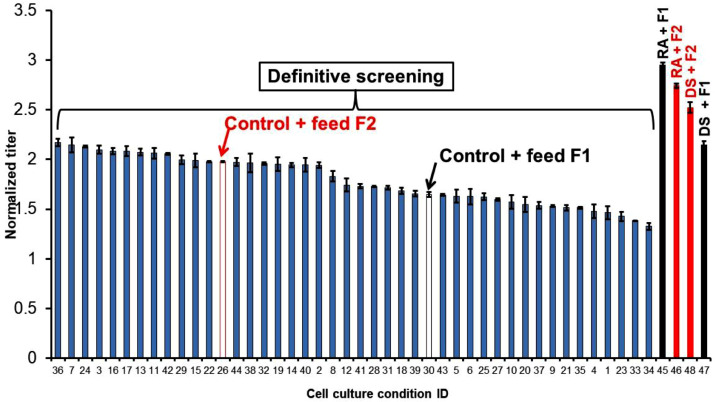
Definitive screening design results for 20 medium components (Table 6) in HTS4 using B1 basal and either F1 or F2 feed. Four conditions with the addition of either RA or dextran sulfate (DS) were used as references (*n* = 2). All raw data for HTS4 are presented in Supplemental Table 2.

Since all of the polyphenolic compounds were new raw materials and dextran sulfate was not a preferred raw material in manufacturing, HTS4 was performed using a definitive screening design with 20 GMP qualified medium additives in one of two different in-house feeds (i.e. F1 or F2 feed) yielding 44 total conditions (Table 6). For comparison, four other conditions were also run in HTS4 with either RA, the most promising polyphenolic flavonoid from HTS3, or dextran sulfate (i.e. RA+F1, RA+F2, DS+F1 and DS+F2 in Fig. 3). Although some of the conditions in the definitive screening design achieved a higher titer than the control conditions, both RA and dextran sulfate conditions had higher titers (Fig. 3). Therefore, despite extensive screening of other media additives, RA and dextran sulfate had the most significant positive effects on titer, demonstrating the advantage of incorporating RA and dextran sulfate into the late phase process development. In particular, RA was the most effective polyphenolic flavonoid on increasing titer (Fig. 2). It was also observed that RA achieved a better titer in conditions with F1 than F2, while dextran sulfate achieved better titer in conditions with F2 than F1 (Fig. 3). F1 is a richer feed with higher concentrations of amino acids and lipids than F2. While not studied further here, this suggests potential interactions between RA or dextran sulfate with lipids or amino acids, and, more generally, that the effects of RA and dextran sulfate were dependent on the overall composition of the feed.

**Table 6:** Medium factors tested in HTS4 cell culture using definitive screening design for 44 conditions in 50 mL TubeSpin bioreactors. DPM2 refers to a proprietary dry media powder added to the feed.

ID	Factors	Level 1	Level 2	Level 3
M1	Feed Media	F2	F1	N/A
M2	Lipoic Acid	-1	0	1
M3	Monothioglycerol	-1	0	1
M4	Putrescine	-1	0	1
M5	Spermine	-1	0	1
M6	Ethanolamine	-1	0	1
M7	Thiamine	-1	0	1
M8	Choline Chloride	-1	0	1
M9	Niacinamide	-1	0	1
M10	Pyridoxal	-1	0	1
M11	Riboflavin	-1	0	1
M12	L-Aspartic acid	-1	0	1
M13	L-Lysine	-1	0	1
M14	L-Tyrosine	-1	0	1
M15	Valine	-1	0	1
M16	Na2HPO4	-1	0	1
M17	MgSO4	-1	0	1
M18	ZnSO4	-1	0	1
M19	CuSO4	-1	0	1
M20	DPM2	-1	0	1

## 2 Experimental Design, Materials and Methods

### 2.1 HTS fed-batch cell culture

JMP (SAS) was used to design and analyze the custom DOE and definitive screening designs of the HTS cell cultures. These designs tested the proprietary chemically defined basal and feed media types, media components, and additives. Fed-batch production culture was performed in 50 mL TubeSpin bioreactors inoculated at the seeding density 0.6 × 106 cells/mL. The cells used to inoculate the production cultures were derived from a working cell bank vial that was expanded for a total 6–12 passages, each passage lasting 3 days. The TubeSpin tubes were incubated in the incubator (Infors, Laurel, MD) at 300 rpm, 5% CO2, 80% humidity, and a temperature of 37 °C, shifted to 35 °C when viable cell density (VCD) reached ≥10 × 106 cells/mL. Feeding was started when an appropriate VCD was reached and continued daily thereafter with a total 40–50% of the initial working volume at 18 mL. The culture was harvested on day 14. The Tecan handler (Tecan, Männedorf, Switzerland) was used for inoculation, sampling and feeding operations.

### 2.2 Titer assay

To quantify the titers in the HTS cultures, 10 mL of day 14 sample was centrifuged at 1000 g for 10 min, filtered with syringe filters (25 mm, 0.08/0.2, Acrodisc, 4187, Pall), and run through a Protein A affinity column (POROS A20 2.1 × 30 mm column, Life Technologies Corporation, Bedford, MA). HPLC (Waters, Milford, MA) was performed using a 2695 Separation Module, 2487 or 2489 Dual Wavelength Detector, and Empower 2 software, a procedure developed by Bristol Myers Squibb. The eluted mAb titer was measured by absorbance at 280 nm and quantified by comparing with the reference standards [2].

## Ethics Statement

This work did not involve the use of human subjects or animal experiments.

## CRediT authorship contribution statement

**Qin He:** Writing – original draft, Investigation, Data curation, Methodology. **Jianlin Xu:** Conceptualization, Investigation, Methodology, Data curation, Writing – review & editing. **Erik Vandermark:** Investigation, Data curation, Methodology, Writing – review & editing. **Jun Tian:** Conceptualization, Investigation, Methodology, Data curation. **Yueming Qian:** Writing – review & editing. **Cynthia Chelius:** Writing – review & editing. **Jongchan Lee:** Writing – review & editing. **Michael C. Borys:** Writing – review & editing. **Zheng Jian Li:** Supervision. **Girish Pendse:** Supervision.

## Declaration of Competing Interest

The authors declare that they have no known competing financial interests or personal relationships that could have appeared to influence the work reported in this paper.

## Appendix Supplementary materials

Image, application 1

## References

[1] Xu J., Rehmann M.S., Tian J., He Q., Chen J., Lee J., Borys M.C., Li Z.J. (2020). Rosmarinic acid, a new raw material, doubled monoclonal antibody titer in cell culture manufacturing. Biochem. Eng. J..

[2] Xu J., Rehmann M.S., Xu M., Zheng S., Hill C., He Q., Borys M.C., Li Z.J. (2020). Development of an intensified fed-batch production platform with doubled titers using N-1 perfusion seed for cell culture manufacturing. Bioresour. Bioprocess..

[3] J. Tian, J. Xu, Q. He (2020) Use of phenolic antioxidants in cell bioproduction. US patent application 20200172949.

