# Supplementary material for: High throughput screening data for a case study of CHO cell culture process development
Source: Data Brief. 2021 Oct 17;39:107491. doi: 10.1016/j.dib.2021.107491 (PMC8531557; doi:10.1016/j.dib.2021.107491)
Supplement: Supplementary file 1 [file mmc1.docx]

**Supplemental Table 1** Raw data for effects of polyphenolic flavonoids, e.g., catechin (Cat), resveratrol (Res) and RA, and different dextran sulfate (DS) products on the mAb titer in HTS3 using OFAT design with B1 basal and F2 feed (n=2).

| **Run#** | **Normalized titer** | | |
| --- | --- | --- | --- |
|  | **A** | **B** | **Mean** |
| Control-B1-F2 | 1.77 | 1.67 | 1.72 |
| Cat-0.025B1-0.25F2 | 2.62 | 2.10 | 2.36 |
| Cat-0.1B1-1F2 | 2.84 | 2.60 | 2.72 |
| Res-0.025B1-0.25F2 | 2.37 | 2.15 | 2.26 |
| Res-0.1B1-1F2 | 2.77 | 2.62 | 2.69 |
| RA-0.025B1-0.25F2 | 3.02 | 2.94 | 2.98 |
| RA-0.1B1-1F2 | 3.21 | 3.30 | 3.25 |
| DS1-50shot | 2.51 | 2.48 | 2.50 |
| DS1-50F2 | 2.24 | 2.09 | 2.16 |
| DS1-100F2 | 2.40 | 2.40 | 2.40 |
| DS1-150F2 | 2.34 | 2.46 | 2.40 |
| DS2-50shot | 2.43 | 2.54 | 2.49 |
| DS2-50F2 | 1.98 | 1.88 | 1.93 |
| DS2-100F2 | 2.19 | 2.10 | 2.14 |
| DS2-150F2 | 2.31 | 2.06 | 2.19 |
| DS3-50shot | 2.32 | 2.25 | 2.28 |
| DS3-50F2 | 1.70 | 1.59 | 1.64 |
| DS3-100F2 | 1.66 | 1.58 | 1.62 |
| DS3-150F2 | 1.78 | 1.68 | 1.73 |
| DS4-50shot | 2.15 | 2.15 | 2.15 |
| DS4-50F2 | 1.79 | 1.79 | 1.79 |
| DS4-100F2 | 1.86 | 1.79 | 1.83 |
| DS4-150F2 | 1.95 | 1.81 | 1.88 |
| DS5-50F2 | 1.76 | 1.59 | 1.67 |
| DS5-100F2 | 1.77 | 1.63 | 1.70 |
| DS5-150F2 | 1.78 | 1.65 | 1.72 |
| DS6-50F2 | 1.80 | 1.66 | 1.73 |
| DS6-100F2 | 1.72 | 1.57 | 1.65 |
| DS6-150F2 | 1.61 | 1.61 | 1.61 |
| DS7-50F2 | 1.73 | 1.64 | 1.68 |
| DS7-100F2 | 1.76 | 1.53 | 1.64 |
| DS7-150F2 | 1.82 | 1.65 | 1.74 |

**Supplemental Table 2** Raw data for definitive screening design for 20 medium components (e.g., M1, M2, etc, as shown in Table 6) in HTS4 using B1 basal and either F1 or F2 feed. Four conditions with the addition of either RA or dextran sulfate (DS) were used as references (n=2).

| Run# | M1 | M2 | M3 | M4 | M5 | M6 | M7 | M8 | M9 | M10 | M11 | M12 | M13 | M14 | M15 | M16 | M17 | M18 | M19 | M20 | RA | DS | Normalized titer | | |
| --- | --- | --- | --- | --- | --- | --- | --- | --- | --- | --- | --- | --- | --- | --- | --- | --- | --- | --- | --- | --- | --- | --- | --- | --- | --- |
|  |  |  |  |  |  |  |  |  |  |  |  |  |  |  |  |  |  |  |  |  |  |  | A | B | Mean |
| 1 | F1 | 1 | -1 | -1 | 1 | 0 | -1 | 1 | 1 | -1 | -1 | -1 | -1 | 1 | -1 | 1 | -1 | 1 | 1 | 1 | N/A | N/A | 1.41 | 1.52 | 1.46 |
| 2 | F2 | 1 | -1 | -1 | 1 | -1 | 1 | -1 | 1 | 1 | 1 | 1 | -1 | -1 | 1 | 0 | -1 | 1 | 1 | -1 | N/A | N/A | 1.92 | 1.97 | 1.94 |
| 3 | F2 | 1 | 1 | -1 | 1 | 1 | 1 | 1 | -1 | -1 | 1 | 0 | -1 | 1 | 1 | -1 | -1 | -1 | -1 | 1 | N/A | N/A | 2.07 | 2.13 | 2.10 |
| 4 | F1 | -1 | 1 | 1 | -1 | 1 | -1 | 1 | -1 | -1 | -1 | -1 | 1 | 1 | -1 | 0 | 1 | -1 | -1 | 1 | N/A | N/A | 1.42 | 1.53 | 1.48 |
| 5 | F1 | 0 | 0 | 0 | 0 | 0 | 0 | 0 | 0 | 0 | 0 | 0 | 0 | 0 | 0 | 0 | 0 | 0 | 0 | 0 | N/A | N/A | 1.58 | 1.68 | 1.63 |
| 6 | F1 | 0 | 0 | 0 | 0 | 0 | 0 | 0 | 0 | 0 | 0 | 0 | 0 | 0 | 0 | 0 | 0 | 0 | 0 | 0 | N/A | N/A | 1.57 | 1.69 | 1.63 |
| 7 | F2 | -1 | 1 | -1 | 1 | -1 | -1 | -1 | -1 | 1 | 1 | -1 | 0 | 1 | -1 | -1 | 1 | 1 | 1 | 1 | N/A | N/A | 2.09 | 2.20 | 2.14 |
| 8 | F1 | -1 | -1 | -1 | -1 | -1 | 1 | 1 | -1 | 0 | 1 | -1 | -1 | 1 | 1 | 1 | 1 | -1 | 1 | -1 | N/A | N/A | 1.79 | 1.87 | 1.83 |
| 9 | F1 | 0 | 1 | 1 | 1 | 1 | 1 | 1 | 1 | 1 | 1 | 1 | 1 | 1 | 1 | 1 | 1 | 1 | 1 | 1 | N/A | N/A | 1.52 | 1.54 | 1.53 |
| 10 | F1 | 1 | -1 | 1 | -1 | 1 | 1 | 1 | 1 | -1 | -1 | 1 | 0 | -1 | 1 | 1 | -1 | -1 | -1 | -1 | N/A | N/A | 1.52 | 1.63 | 1.57 |
| 11 | F2 | 1 | 1 | -1 | 1 | -1 | 1 | 1 | 1 | 1 | -1 | -1 | 1 | 0 | -1 | 1 | 1 | -1 | -1 | -1 | N/A | N/A | 2.02 | 2.10 | 2.06 |
| 12 | F1 | 1 | -1 | 1 | 1 | 1 | 1 | -1 | -1 | 1 | 0 | -1 | 1 | 1 | -1 | -1 | -1 | -1 | 1 | -1 | N/A | N/A | 1.69 | 1.79 | 1.74 |
| 13 | F2 | 1 | -1 | 1 | -1 | 1 | -1 | 1 | 1 | 1 | 1 | -1 | -1 | 1 | 0 | -1 | 1 | 1 | -1 | -1 | N/A | N/A | 2.05 | 2.10 | 2.07 |
| 14 | F2 | 0 | -1 | -1 | -1 | -1 | -1 | -1 | -1 | -1 | -1 | -1 | -1 | -1 | -1 | -1 | -1 | -1 | -1 | -1 | N/A | N/A | 1.93 | 1.96 | 1.95 |
| 15 | F2 | 1 | 1 | 1 | 1 | 1 | -1 | -1 | 1 | 0 | -1 | 1 | 1 | -1 | -1 | -1 | -1 | 1 | -1 | 1 | N/A | N/A | 1.94 | 2.04 | 1.99 |
| 16 | F2 | 1 | -1 | -1 | -1 | 1 | -1 | 1 | -1 | 1 | 1 | 1 | 1 | -1 | -1 | 1 | 0 | -1 | 1 | 1 | N/A | N/A | 2.05 | 2.11 | 2.08 |
| 17 | F2 | -1 | -1 | -1 | -1 | 1 | 1 | -1 | 0 | 1 | -1 | -1 | 1 | 1 | 1 | 1 | -1 | 1 | -1 | 1 | N/A | N/A | 2.04 | 2.12 | 2.08 |
| 18 | F1 | -1 | -1 | 1 | -1 | -1 | -1 | -1 | 1 | 1 | -1 | 0 | 1 | -1 | -1 | 1 | 1 | 1 | 1 | -1 | N/A | N/A | 1.66 | 1.71 | 1.69 |
| 19 | F2 | 1 | 1 | 1 | -1 | -1 | -1 | -1 | 1 | -1 | 1 | -1 | 1 | 1 | 1 | 1 | -1 | -1 | 1 | 0 | N/A | N/A | 1.90 | 2.00 | 1.95 |
| 20 | F1 | 1 | 1 | -1 | -1 | 1 | 0 | -1 | 1 | 1 | -1 | -1 | -1 | -1 | 1 | -1 | 1 | -1 | 1 | 1 | N/A | N/A | 1.49 | 1.60 | 1.55 |
| 21 | F1 | 1 | -1 | 1 | 1 | -1 | -1 | -1 | -1 | 1 | -1 | 1 | -1 | 1 | 1 | 1 | 1 | -1 | -1 | 1 | N/A | N/A | 1.49 | 1.54 | 1.52 |
| 22 | F2 | -1 | 1 | 1 | -1 | 0 | 1 | -1 | -1 | 1 | 1 | 1 | 1 | -1 | 1 | -1 | 1 | -1 | -1 | -1 | N/A | N/A | 1.97 | 1.99 | 1.98 |
| 23 | F1 | 1 | -1 | -1 | -1 | -1 | 1 | -1 | 1 | -1 | 1 | 1 | 1 | 1 | -1 | -1 | 1 | 0 | -1 | 1 | N/A | N/A | 1.39 | 1.46 | 1.43 |
| 24 | F2 | -1 | -1 | -1 | 1 | 1 | -1 | 0 | 1 | -1 | -1 | 1 | 1 | 1 | 1 | -1 | 1 | -1 | 1 | -1 | N/A | N/A | 2.12 | 2.14 | 2.13 |
| 25 | F1 | -1 | 1 | -1 | -1 | 1 | 1 | 1 | 1 | -1 | 1 | -1 | 1 | -1 | -1 | -1 | -1 | 1 | 1 | -1 | N/A | N/A | 1.60 | 1.65 | 1.63 |
| 26 | F2 | -1 | 1 | -1 | -1 | -1 | -1 | 1 | 1 | -1 | 0 | 1 | -1 | -1 | 1 | 1 | 1 | 1 | -1 | 1 | N/A | N/A | 1.97 | 1.98 | 1.98 |
| 27 | F1 | 1 | 1 | 1 | 1 | -1 | -1 | 1 | 0 | -1 | 1 | 1 | -1 | -1 | -1 | -1 | 1 | -1 | 1 | -1 | N/A | N/A | 1.59 | 1.61 | 1.60 |
| 28 | F1 | 1 | 1 | -1 | -1 | -1 | -1 | 1 | -1 | 1 | -1 | 1 | 1 | 1 | 1 | -1 | -1 | 1 | 0 | -1 | N/A | N/A | 1.72 | 1.74 | 1.73 |
| 29 | F2 | -1 | 1 | 1 | 1 | 1 | -1 | 1 | -1 | 1 | -1 | -1 | -1 | -1 | 1 | 1 | -1 | 0 | 1 | -1 | N/A | N/A | 1.96 | 2.03 | 2.00 |
| 30 | F1 | -1 | -1 | 0 | 1 | -1 | -1 | 1 | 1 | 1 | 1 | -1 | 1 | -1 | 1 | -1 | -1 | -1 | -1 | 1 | N/A | N/A | 1.62 | 1.67 | 1.65 |
| 31 | F1 | -1 | 1 | -1 | 0 | 1 | -1 | -1 | 1 | 1 | 1 | 1 | -1 | 1 | -1 | 1 | -1 | -1 | -1 | -1 | N/A | N/A | 1.70 | 1.73 | 1.72 |
| 32 | F2 | 1 | 1 | 0 | -1 | 1 | 1 | -1 | -1 | -1 | -1 | 1 | -1 | 1 | -1 | 1 | 1 | 1 | 1 | -1 | N/A | N/A | 1.95 | 1.97 | 1.96 |
| 33 | F1 | -1 | -1 | 1 | -1 | 1 | -1 | -1 | -1 | -1 | 1 | 1 | -1 | 0 | 1 | -1 | -1 | 1 | 1 | 1 | N/A | N/A | 1.37 | 1.39 | 1.38 |
| 34 | F1 | -1 | 1 | -1 | 1 | -1 | 1 | -1 | -1 | -1 | -1 | 1 | 1 | -1 | 0 | 1 | -1 | -1 | 1 | 1 | N/A | N/A | 1.30 | 1.36 | 1.33 |
| 35 | F1 | 1 | 1 | 1 | -1 | -1 | 1 | 0 | -1 | 1 | 1 | -1 | -1 | -1 | -1 | 1 | -1 | 1 | -1 | 1 | N/A | N/A | 1.50 | 1.52 | 1.51 |
| 36 | F2 | -1 | 0 | 1 | -1 | -1 | 1 | 1 | 1 | 1 | -1 | 1 | -1 | 1 | -1 | -1 | -1 | -1 | 1 | 1 | N/A | N/A | 2.14 | 2.20 | 2.17 |
| 37 | F1 | 1 | 0 | -1 | 1 | 1 | -1 | -1 | -1 | -1 | 1 | -1 | 1 | -1 | 1 | 1 | 1 | 1 | -1 | -1 | N/A | N/A | 1.51 | 1.57 | 1.54 |
| 38 | F2 | 1 | -1 | 1 | 0 | -1 | 1 | 1 | -1 | -1 | -1 | -1 | 1 | -1 | 1 | -1 | 1 | 1 | 1 | 1 | N/A | N/A | 1.89 | 2.04 | 1.97 |
| 39 | F1 | -1 | 1 | 1 | 1 | -1 | 1 | -1 | 1 | -1 | -1 | -1 | -1 | 1 | 1 | -1 | 0 | 1 | -1 | -1 | N/A | N/A | 1.63 | 1.68 | 1.66 |
| 40 | F2 | -1 | -1 | 1 | 1 | 1 | 1 | -1 | 1 | -1 | 1 | -1 | -1 | -1 | -1 | 1 | 1 | -1 | 0 | 1 | N/A | N/A | 1.89 | 2.00 | 1.95 |
| 41 | F1 | -1 | -1 | -1 | 1 | 1 | 1 | 1 | -1 | 1 | -1 | 1 | -1 | -1 | -1 | -1 | 1 | 1 | -1 | 0 | N/A | N/A | 1.71 | 1.75 | 1.73 |
| 42 | F2 | -1 | -1 | 1 | 1 | -1 | 0 | 1 | -1 | -1 | 1 | 1 | 1 | 1 | -1 | 1 | -1 | 1 | -1 | -1 | N/A | N/A | 2.04 | 2.07 | 2.06 |
| 43 | F1 | -1 | -1 | -1 | -1 | -1 | -1 | -1 | -1 | -1 | -1 | -1 | -1 | -1 | -1 | -1 | -1 | -1 | -1 | -1 | N/A | N/A | 1.63 | 1.66 | 1.64 |
| 44 | F2 | -1 | -1 | -1 | -1 | -1 | -1 | -1 | -1 | -1 | -1 | -1 | -1 | -1 | -1 | -1 | -1 | -1 | -1 | -1 | N/A | N/A | 1.94 | 2.00 | 1.97 |
| 45 | F1 | -1 | -1 | -1 | -1 | -1 | -1 | -1 | -1 | -1 | -1 | -1 | -1 | -1 | -1 | -1 | -1 | -1 | -1 | -1 | RA | N/A | 2.93 | 2.97 | 2.95 |
| 46 | F2 | -1 | -1 | -1 | -1 | -1 | -1 | -1 | -1 | -1 | -1 | -1 | -1 | -1 | -1 | -1 | -1 | -1 | -1 | -1 | RA | N/A | 2.72 | 2.76 | 2.74 |
| 47 | F1 | -1 | -1 | -1 | -1 | -1 | -1 | -1 | -1 | -1 | -1 | -1 | -1 | -1 | -1 | -1 | -1 | -1 | -1 | -1 | N/A | DS | 2.11 | 2.18 | 2.15 |
| 48 | F2 | -1 | -1 | -1 | -1 | -1 | -1 | -1 | -1 | -1 | -1 | -1 | -1 | -1 | -1 | -1 | -1 | -1 | -1 | -1 | N/A | DS | 2.48 | 2.56 | 2.52 |
